# Serodiagnosis of Zika virus (ZIKV) infections by a novel NS1-based ELISA devoid of cross-reactivity with dengue virus antibodies: a multicohort study of assay performance, 2015 to 2016

**DOI:** 10.2807/1560-7917.ES.2016.21.50.30426

**Published:** 2016-12-15

**Authors:** Katja Steinhagen, Christian Probst, Christiane Radzimski, Jonas Schmidt-Chanasit, Petra Emmerich, Marjan van Esbroeck, Janke Schinkel, Martin P Grobusch, Abraham Goorhuis, Jens M Warnecke, Erik Lattwein, Lars Komorowski, Andrea Deerberg, Sandra Saschenbrecker, Winfried Stöcker, Wolfgang Schlumberger

**Affiliations:** 1 Institute for Experimental Immunology, EUROIMMUN AG, Lübeck, Germany; 2 WHO Collaborating Centre for Arbovirus and Haemorrhagic Fever Reference and Research, Bernhard-Nocht Institute for Tropical Medicine, Hamburg, Germany; 3 German Center for Infection Research (DZIF), Partner Site Hamburg-Lübeck-Borstel, Hamburg, Germany; 4 National Reference Center for Arboviruses, Department of Clinical Sciences, Institute of Tropical Medicine, Antwerp, Belgium; 5 Department of Medical Microbiology, Section of Clinical Virology, Academic Medical Center, Public Health Service, Amsterdam, the Netherlands; 6 Center for Tropical Medicine and Travel Medicine, Department of Infectious Diseases, Division of Internal Medicine, Academic Medical Center, University of Amsterdam, Amsterdam, the Netherlands; 7 Institute of Tropical Medicine, University of Tübingen, Tübingen, Germany

**Keywords:** Zika virus, non-structural protein 1, NS1, antibody, ELISA, infection

## Abstract

Serological diagnosis of Zika virus (ZIKV) infections is challenging due to high cross-reactivity between flaviviruses. We evaluated the diagnostic performance of a novel anti-ZIKV ELISA based on recombinant ZIKV non-structural protein 1 (NS1). Assay sensitivity was examined using sera from 27 patients with reverse transcription (RT)-PCR-confirmed and 85 with suspected ZIKV infection. Specificity was analysed using sera from 1,015 healthy individuals. Samples from 252 patients with dengue virus (n = 93), West Nile virus (n = 34), Japanese encephalitis virus (n = 25), chikungunya virus (n = 19) or *Plasmodium* spp. (n = 69) infections and from 12 yellow fever-vaccinated individuals were also examined. In confirmed ZIKV specimens collected ≥ 6 days after symptom onset, ELISA sensitivity was 58.8% (95% confidence interval (CI): 36.0–78.4) for IgM, 88.2% (95% CI: 64.4–98.0) for IgG, and 100% (95% CI: 78.4–100) for IgM/IgG, at 99.8% (95% CI: 99.2–100) specificity. Cross-reactivity with high-level dengue virus antibodies was not detected. Among patients with potentially cross-reactive antibodies anti-ZIKV positive rates were 0.8% (95% CI: 0–3.0) and 0.4% (95% CI: 0–2.4) for IgM and IgG, respectively. Providing high specificity and low cross-reactivity, the NS1-based ELISA has the potential to aid in counselling patients, pregnant women and travellers after returning from ZIKV-endemic areas.

## Introduction

Zika virus (ZIKV) is an emerging mosquito-transmitted flavivirus currently causing large epidemics in South and Central America as well as in the Caribbean, presenting a global public health emergency [1]. It is closely related to other human pathogenic members of the flavivirus family such as dengue virus (DENV), West Nile virus (WNV), Japanese encephalitis virus (JEV) and yellow fever virus (YFV). Besides their structural resemblance, most of these viruses share a partially overlapping geographical distribution, with tropical and subtropical regions representing the favourable environment of the main vector, mosquitos of the genus *Aedes* [2].

The diagnosis of ZIKV infections is increasingly relevant for European countries where, up to now, only a small number of travellers returning from endemic areas have contracted the virus [3]. However, there are concerns that ZIKV might be imported by infected individuals and spread through sexual transmission and via *Aedes* mosquitos that are endemic in parts of southern Europe.

The clinical symptoms associated with ZIKV infection include fever, rash, arthralgia, myalgia and conjunctivitis, and are normally self-limiting. The proportion of asymptomatic ZIKV infections is still unknown, but there is evidence that infection may go unrecognised in a considerable number of cases [1,4]. In the acute phase, fever due to ZIKV infection is difficult to differentiate clinically from that due to DENV infections [5]. Chikungunya virus (CHIKV), belonging to the *Togaviridae* family, should also be considered in differential diagnostics, as it is transmitted by the same mosquito vector and circulates in the same regions [2]. The common distribution and similar clinical presentation, in combination with high variation in disease outcome of ZIKV-, DENV- and CHIKV-infected patients, highlight the need for specific and reliable diagnostic methods. Knowing the infecting virus can be of clinical relevance, for example, when ZIKV infection is suspected in women during pregnancy, which could result in fetal malformations, or in men who could transmit the virus sexually, or, in cases of CHIKV infection with prolonged arthralgias, where correct diagnosis can help avoid unnecessary rheumatological analysis.

The current ZIKV epidemic, particularly in Brazil, has revealed two potential complications in ZIKV infections, which were initially suspected during the 2007 outbreak in Micronesia [6]. Firstly, a large rise in the number of cases of Guillain–Barré syndrome (GBS), an autoimmune disease resulting from damage of peripheral-nerve myelin, was triggered by ZIKV infections [1,7]. Secondly, a strong causative link was suggested between fetal abnormalities and ZIKV infection during early pregnancy, based on a 20-fold increase in newborn microcephaly in highly endemic regions in Brazil, followed by the first reports of ZIKV genome detection in amniotic fluid and fetal brain after intrauterine diagnosis of microcephaly [1,8-10].

At present, diagnosis of ZIKV infections is challenging because the only specific tool is direct virus detection using nucleic acid-based testing (NAT), with ZIKV RNA detectable in serum up to 7 days after symptom onset and even longer in saliva, urine (about 20 days) and semen (> 20 days) [6,11-13]. Plaque-reduction neutralisation tests (PRNTs) can measure virus-specific neutralising antibodies, a fact that is relevant in regions where two or more flaviviruses co-occur. However, PRNTs do not discriminate between antibody classes and, especially in secondary flavivirus infections, cross-reactive neutralising antibodies may contribute to virus neutralisation [6,14,15], thus questioning the suitability of PRNTs for the confirmation of active infection. In addition, PRNTs are time-consuming, difficult to perform, not suitable for testing large panels, and therefore restricted to highly specialised laboratories. In contrast, ELISA-based measurement is a rapid, scalable and technically mature approach. IgM antibodies against flavivirus antigens are first produced 4 to 7 days after infection, and IgG antibodies appear a few days later. However, a major limitation of current ELISAs for diagnosing flaviviral infections is their extensive cross-reactivity within the *Flavivirus* genus [6].

The molecular organisation of flaviviruses is conserved. Virions consist of single-stranded positive RNA surrounded by an icosahedral capsid and envelope. The RNA encodes for a single polyprotein, which is processed into structural (C, prM, and E) and non-structural (NS1 to NS5) proteins [16]. Knowledge about NS1 is mainly derived from the well-studied flaviviruses (DENV, WNF, YFV), whereas little is known about NS1 from ZIKV. NS1 proteins (molecular mass 46–55 kDa) are present in two distinct variants [17]. Membrane-associated NS1 is mainly found as a dimer that interacts with intracellular membranes, such as the endoplasmic reticulum and vesicle packets, and with the cell surface [18,19]. Secreted NS1 assembles into barrel-shaped hexamers consisting of three dimers [20,21]. The NS1 function remains elusive, although roles in RNA replication [18], intracellular protein transport, virion release [22] and immunomodulatory activities [17] have been proposed. As reported for DENV and WNV, NS1 is secreted by infected cells into the bloodstream [23,24], stimulating the immune system to produce high NS1 antibody titres. However, acute-phase release of ZIKV-NS1 into patient’s serum has not yet been verified, and a ZIKV-NS1 antigen assay is currently not available [25]. Recombinant NS1 proteins were used in a multiplex serological protein microarray for the detection of anti-DENV, -WNV, and -JEV IgM and IgG, demonstrating high sensitivity and limited cross-reactivity, suggesting NS1 may represent an efficient antigenic substrate [26].

Recently, an ELISA based on recombinant ZIKV-NS1 has been developed [27]. Here, the diagnostic performance of this assay was examined using sera from returning travellers and patients from ZIKV-endemic areas with laboratory-confirmed ZIKV infection, potentially cross-reactive samples from patients with flaviviral and other infections, as well as control panels from blood donors of different ages and geographical origin.

## Methods

### Human sera

The study included serum samples from 27 patients who had tested positive for ZIKV RNA by reverse transcription PCR (RT-PCR); Group 1: travellers returning from endemic areas (n = 8); Group 2: residents in ZIKV-endemic areas (n = 19). On the basis of direct detection of the pathogen’s genome, these cases were referred to as having RT-PCR-confirmed ZIKV infection. Samples from a further 85 patients had been pre-characterised by anti-ZIKV indirect immunofluorescence assay (IIFA; EUROIMMUN, Lübeck, Germany) based on whole virus antigen, showing reactivity for anti-ZIKV IgM and/or IgG; Group 3: travellers returning from endemic areas (n = 26); Group 4: residents in ZIKV-endemic areas (n = 59). Since false-positive results due to cross-reactivity of this IIFA cannot be excluded, these cases were referred to as having suspected ZIKV infection (Table 1).

**Table 1:** Characteristics of patients with RT-PCR-confirmed (n = 27) and suspected (n = 85) Zika virus infection, study evaluating a novel NS1-based ELISA, Germany 2016

Case ID	Age groups in years	Sex	Country of infection	Current/former residence	Sampling Dpso	Phase of infection^a^	Clinical symptoms^b^	Diagnostic centre/ provider of samples	ZIKV-RT-PCR assay/ performed at	ZIKV-RT-PCR result^c^	Virus neutralisation assay titre	IIFA IgM titre^d^	IIFA IgG titre^d^	
Group 1: RT-PCR-confirmed ZIKV infection, travellers returning from ZIKV-endemic areas (n = 8)													
1	20–29	M	NA	Non-endemic	7	Active	Yes	WHOCC, Hamburg, Germany	RealStar Zika Virus RT-PCR (Altona Diagnostics, Hamburg, Germany)/ WHOCC	**Pos**	NA	**1:3,200**	**1:3,200**	
2	30–39	F	Haiti	Non-endemic	≥ 4	Active	Yes			**Pos**	NA	**1:320**	**1:32,000**	
3	50–59	M	NA	Non-endemic	3	Initial	No			**Pos**	NA	NA	NA	
4	50–59	F	NA	Non-endemic	< 4	Initial	NA			**Pos**	NA	**1:100**	**1:1,000**	
5	20–29	F	NA	Non-endemic	17	Active	NA	ITM, Antwerp, Belgium	RealStar Zika Virus RT-PCR (Altona Diagnostics, Hamburg, Germany)/ ITM	**Pos**	**> 1:640**	NA	NA	
6	40–49	M	NA	Non-endemic	11	Active	NA			**Pos**	**1:243**	NA	NA	
7	0–9	M	NA	Non-endemic	3	Initial	NA			**Pos**	NA	NA	NA	
8	20–29	F	NA	Non-endemic	11	Active	NA			**Pos**	**1:788**	NA	NA	
Group 2: RT-PCR-confirmed ZIKV infection, residents in ZIKV-endemic areas (n = 19)														
1	60–69	F	Suriname	The Netherlands/Suriname^e^	3	Initial	NA	AMC, Amsterdam, the Netherlands	In-house Zika RT-PCR/AMC	**Pos**	NA	NA	NA	
2	50–59	M	Suriname	The Netherlands/Suriname^e^	5	Initial	NA			**Pos**	NA	NA	NA	
3	40–49	F	Suriname	The Netherlands/Suriname^e^	11	Active	NA			**Pos**	NA	NA	NA	
4	40–49	M	Suriname	The Netherlands/Suriname^e^	9	Active	NA			**Pos**	NA	NA	NA	
5	50–59	F	Suriname	The Netherlands/Suriname^e^	6	Active	NA			**Pos**	NA	NA	NA	
6	50–59	M	Suriname	The Netherlands/Suriname^e^	6	Active	NA			**Pos**	NA	NA	NA	
7	50–59	F	Suriname	The Netherlands/Suriname^e^	53	Late	NA			**Pos**	NA	NA	NA	
8	50–59	F	Suriname	The Netherlands/Suriname^e^	17	Active	NA			**Pos**	NA	NA	NA	
9	60–69	F	Suriname	The Netherlands/Suriname^e^	24	Late	NA			**Pos**	NA	NA	NA	
10	70–79	M	Suriname	The Netherlands/Suriname^e^	6	Active	NA			**Pos**	NA	NA	NA	
11	0–9	M	Dominican Republic	The Netherlands	1	Initial	NA			**Pos**	NA	NA	NA	
12	50–59	F	Dominican Republic	Dominican Republic	20	Active	Yes	Boca Biolistics, Coconut Creek, Florida, US	Trioplex real-time RT-PCR (CDC, Atlanta, Georgia, US)/CDC	**Pos**	NA	0	**1:32,000**	
13	50–59	F	Dominican Republic	Dominican Republic	31	Late	Yes			**Pos**	NA	**1:100**	**1:32,000**	
14	20–29	M	Colombia	Colombia	3	Initial	Yes	Allied Research Society, Miami Lakes, Florida, US	Trioplex real-time RT-PCR (CDC, Atlanta, Georgia, US)/CDC	**Pos**	NA	0	**1:1,000**	
15	40–49	F	Colombia	Colombia	5	Initial	Yes			**Pos**	NA	0	**1:1,000**	
16	50–59	F	Colombia	Colombia	4	Initial	Yes			**Pos**	NA	**1:10**	**1:3,200**	
17	10–19	M	Colombia	Colombia	3	Initial	Yes			**Pos**	NA	0	**1:3,200**	
18	20–29	F	Colombia	Colombia	6	Active	Yes	Biomex GmbH, Heidelberg, Germany	RealStar Zika Virus RT-PCR (Altona Diagnostics, Hamburg, Germany)/ Altona Diagnostics	**Pos**	NA	**1:3,200**	**1:32,000**	
19	10–19	M	Colombia	Colombia/US	15	Active	Yes		Trioplex real-time RT-PCR (CDC, Atlanta, Georgia, US)/CDC	**Pos**	NA	**1:10**	**1:32,000**	
Group 3: Suspected ZIKV infection, travellers returning from ZIKV-endemic areas (n = 26)													
1	NA	NA	NA	Non-endemic	NA	NA	NA	WHOCC, Hamburg, Germany	NA	NA	NA	**1:3,200**	**1:10,000**	
2	NA	NA	NA	Non-endemic	NA	NA	NA			NA	NA	**1:1,000**	**1:10,000**	
3	NA	NA	NA	Non-endemic	NA	NA	NA			NA	NA	**1:3,200**	**1:10,000**	
4	NA	NA	Brazil	Non-endemic	NA	NA	NA			NA	NA	**1:1,000**	**1:32,000**	
5	NA	NA	Brazil	Non-endemic	NA	NA	NA			NA	NA	**1:1,000**	**1:3,200**	
6	NA	NA	Brazil	Non-endemic	NA	NA	NA			NA	NA	**1:3,200**	**1:10,000**	
7	NA	NA	Brazil	Non-endemic	NA	NA	NA			NA	NA	**1:100**	< 1:100	
8	NA	NA	NA	Non-endemic	NA	NA	NA			NA	NA	**1:1,000**	**1:100**	
9	NA	NA	NA	Non-endemic	NA	NA	NA			NA	NA	**1:320**	**1:10,000**	
10	NA	NA	NA	Non-endemic	NA	NA	NA			NA	NA	**1:320**	**1:32,000**	
11	NA	NA	Brazil	Non-endemic	19	Active	Yes			NA	NA	**1:320**	**1:10,000**	
12	NA	NA	Brazil	Non-endemic	NA	NA	NA			NA	NA	**1:100**	**1:100,000**	
13	NA	NA	Brazil	Non-endemic	NA	NA	NA			NA	NA	**1:1,000**	**1:320**	
14	NA	NA	Brazil	Non-endemic	NA	NA	NA			NA	NA	**1:320**	**1:3,200**	
15	NA	NA	Brazil	Non-endemic	NA	NA	NA			NA	NA	**1:320**	**1:1,000**	
16	NA	NA	Brazil	Non-endemic	NA	NA	NA			NA	NA	**1:1,000**	**1:10,000**	
17	NA	NA	Brazil	Non-endemic	NA	NA	NA			NA	NA	**1:320**	**1:10,000**	
18	NA	NA	NA	Non-endemic	32	Late	NA			NA	NA	**1:100**	**1:32,000**	
19	NA	NA	Colombia	Non-endemic	45	Late	NA			NA	NA	**1:100**	**1:3,200**	
20	NA	NA	NA	Non-endemic	NA	NA	NA			NA	NA	**1:1,000**	**1:10,000**	
21	NA	NA	Denmark	Non-endemic	NA	NA	NA			NA	NA	**1:100**	**1:32,000**	
22	NA	NA	NA	Non-endemic	NA	NA	NA			NA	NA	**1:3,200**	**1:32,000**	
23	NA	NA	Colombia	Non-endemic	NA	NA	NA			NA	NA	**1:100**	**1:10,000**	
24	NA	NA	Brazil	Non-endemic	NA	NA	NA			NA	NA	**1:320**	**1:32,000**	
25	NA	NA	Brazil	Non-endemic	NA	NA	NA			NA	NA	**1:320**	**1:32,000**	
26	NA	NA	Colombia	Non-endemic	15	Active	NA			NA	NA	**1:3,200**	**1:10,000**	
Group 4: Suspected ZIKV infection, residents in ZIKV-endemic areas (n = 59)														
1	30–39	F	Colombia	Colombia	6	Active	Yes	Allied Research Society, Miami Lakes, Florida, US	NA	NA	NA	**1:1,000**	**1:320,000**	
2	20–29	M	Colombia	Colombia	8	Active	Yes			NA	NA	**1:100**	**1:1,000**	
3	30–39	F	Colombia	Colombia	11	Active	Yes			NA	NA	0	**1:1,000**	
4	40–49	M	Colombia	Colombia	14	Active	Yes			NA	NA	**1:3,200**	**1:320,000**	
5	30–39	F	Colombia	Colombia	17	Active	Yes			NA	NA	**1:3,200**	**1:320,000**	
6	80–89	M	Colombia	Colombia	20	Active	Yes			NA	NA	**1:320**	**1:320,000**	
7	50–59	F	Colombia	Colombia	23	Late	Yes			NA	NA	**1:320**	**1:10,000**	
8	30–39	M	Colombia	Colombia	30	Late	Yes			NA	NA	**1:3,200**	**1:320,000**	
9	40–49	F	Colombia	Colombia	49	Late	Yes			NA	NA	**1:100**	**1:10,000**	
10	10–19	F	Colombia	Colombia	54	Late	Yes			NA	NA	**1:10**	**1:1,000**	
11	50–59	F	Colombia	Colombia	6	Active	Yes			NA	NA	0	**1:3,200**	
12	40–49	F	Colombia	Colombia	4	Initial	Yes			NA	NA	0	**1:1,000**	
13	10–19	M	Colombia	Colombia	66	Late	Yes			NA	NA	0	**1:32,000**	
14	40–49	F	Colombia	Colombia	68	Late	Yes			NA	NA	**1:10**	**1:32,000**	
15	50–59	F	NA	Colombia	70	Late	Yes			NA	NA	0	**1:32,000**	
16	40–49	F	NA	Colombia	2	Initial	Yes			NA	NA	0	**1:10,000**	
17	20–29	F	Colombia	Colombia	7	Active	Yes			NA	NA	**1:100**	**1:320,000**	
18	50–59	F	NA	Colombia	4	Initial	Yes			NA	NA	**1:100**	**1:100,000**	
19	40–49	M	Colombia	Colombia	3	Initial	Yes			NA	NA	**1:10,000**	**1:32,000**	
20	40–49	F	Colombia	Colombia	4	Initial	Yes			NA	NA	**1:32**	**1:32,000**	
21	30–39	M	Colombia	Colombia	4	Initial	Yes			NA	NA	**1:32**	**1:32,000**	
22	40–49	F	Colombia	Colombia	4	Initial	Yes			NA	NA	0	**1:100,000**	
23	30–39	M	Colombia	Colombia	4	Initial	Yes			NA	NA	0	**1:32,000**	
24	20–29	F	Colombia	Colombia	5	Initial	Yes			NA	NA	**1:10**	**1:10,000**	
25	40–49	F	Colombia	Colombia	5	Initial	Yes			NA	NA	**1:1,000**	**1:100,000**	
26	30–39	F	Colombia	Colombia	3	Initial	Yes			NA	NA	0	**1:3,200**	
27	40–49	F	Colombia	Colombia	4	Initial	Yes			NA	NA	0	**1:32,000**	
28	20–29	F	Colombia	Colombia	3	Initial	Yes			NA	NA	0	**1:320**	
29	50–59	F	Colombia	Colombia	4	Initial	Yes			NA	NA	0	**1:10,000**	
30	20–29	F	Colombia	Colombia	3	Initial	Yes			NA	NA	**1:32**	**1:10,000**	
31	30–39	F	Colombia	Colombia	3	Initial	Yes	Biomex GmbH, Heidelberg, Germany	NA	NA	NA	0	**1:32,000**	
32	20–29	F	Colombia	Colombia	4	Initial	Yes			NA	NA	**1:100**	**1:32,000**	
33	10–19	F	Colombia	Colombia	9	Active	Yes			NA	NA	**1:100**	**1:32,000**	
34	20–29	F	Colombia	Colombia	12	Active	Yes			NA	NA	0	**1:32,000**	
35	10–19	F	Colombia	Colombia	20	Active	Yes			NA	NA	**1:100**	**1:10,000**	
36	20–29	F	Colombia	Colombia	27	Late	Yes			NA	NA	**1:320**	**1:10,000**	
37	30–39	F	Colombia	Colombia	36	Late	Yes			NA	NA	**1:10**	**1:32,000**	
38	10–19	F	Colombia	Colombia	56	Late	Yes			NA	NA	**1:100**	**1:10,000**	
39	30–39	F	Colombia	Colombia	67	Late	Yes			NA	NA	**1:10**	**1:32,000**	
40	10–19	F	Colombia	Colombia	2	Initial	Yes	Allied Research Society, Miami Lakes, Florida, US	NA	NA	NA	0	**1:10,000**	
41	30–39	F	Colombia	Colombia	5	Initial	Yes			NA	NA	**1:320**	**1:10,000**	
42	20–29	F	Colombia	Colombia	6	Active	Yes			NA	NA	**1:100**	**1:10,000**	
43	20–29	F	Colombia	Colombia	8	Active	Yes			NA	NA	**1:100**	**1:32,000**	
44	30–39	F	Colombia	Colombia	15	Active	Yes			NA	NA	0	**1:10,000**	
45	20–29	F	Colombia	Colombia	21	Late	Yes			NA	NA	**1:10**	**1:100,000**	
46	20–29	F	Colombia	Colombia	29	Late	Yes			NA	NA	**1:320**	**1:32,000**	
47	20–29	F	Colombia	Colombia	38	Late	Yes			NA	NA	**1:1,000**	**1:320,000**	
48	10–19	F	Colombia	Colombia	50	Late	Yes			NA	NA	**1:10**	**1:10,000**	
49	20–29	F	Colombia	Colombia	88	Late	Yes			NA	NA	0	**1:1,000**	
50	40–49	F	Colombia	Colombia	2	Initial	Yes			NA	NA	0	**1:3,200**	
51	20–29	M	Colombia	Colombia	5	Initial	Yes			NA	NA	**1:1,000**	**1:32,000**	
52	30–39	F	Colombia	Colombia	6	Active	Yes			NA	NA	0	**1:1,000**	
53	20–29	M	Colombia	Colombia	8	Active	Yes			NA	NA	0	**1:10,000**	
54	30–39	F	Colombia	Colombia	15	Active	Yes			NA	NA	**1:320**	**1:320,000**	
55	30–39	M	Colombia	Colombia	21	Late	Yes			NA	NA	**1:100**	**1:32,000**	
56	40–49	M	Colombia	Colombia	29	Late	Yes			NA	NA	**1:32,000**	**1:32,000**	
57	40–49	F	Colombia	Colombia	38	Late	Yes			NA	NA	0	**1:320**	
58	50–59	F	Colombia	Colombia	50	Late	Yes			NA	NA	0	**1:100,000**	
59	50–59	M	Colombia	Colombia	85	Late	Yes			NA	NA	0	**1:32,000**	

AMC: Academic Medical Center; CDC: Centers for Disease Control and Prevention; dpso: days post symptom onset; F: female; IIFA: indirect immunofluorescence assay; ITM: Institute of Tropical Medicine, M: male; NA: not available; NS: non-structural protein; Pos: positive; US: United States; WHOCC: World Health Organization Collaborating Centre (for Arbovirus and Haemorrhagic Fever Reference and Research); ZIKV: Zika virus.

^a^ Phase of infection at the time of sample collection: initial phase: ≤ 5 dpso; active phase: 6 to 20 dpso; late phase: > 20 dpso [28].

^b^ Fever, skin rash, joint pain, myalgia, headache, conjunctivitis, eye pain, diarrhoea and malaise.

^c^ ZIKV-RT-PCR results can also refer to serum or urine samples taken at an earlier date than the samples used for anti-ZIKV serological testing.

^d^ IIFA was performed at EUROIMMUN, Lübeck, Germany, using the Anti-Zika Virus IIFA test kit (EUROIMMUN). Cut-off IgM: ≥ 1:10; IgG: ≥ 1:100.

^e^ Sera from Dutch residents who were born and raised in Suriname and/or had visited their country of origin occasionally.

Classification into three stages of ZIKV infection was according to the Pan American Health Organization (PAHO) /World Health Organization (WHO) recommendations on ZIKV surveillance in the Americas [28]: ≤ 5 days post symptom onset, initial stage; 6–20 days post symptom onset, active stage; > 20 days post symptom onset, late stage. Samples from travellers returning from endemic areas were provided by the diagnostic institutes (listed in Table 1) to which they had been sent for routine diagnostic testing. Samples from patients residing in Latin America (i.e. Dominican Republic and Colombia) were purchased from Boca Biolistics (Coconut Creek, Florida, United States (United States (US)), Allied Research Society (Miami Lakes, Florida, US) and Biomex GmbH (Heidelberg, Germany). As confirmed by these institutes and companies, written informed consent had been obtained from all patients, and there were no legal or ethical restrictions to using the samples.

To evaluate cross-reactivity, samples were used from 252 patients with either a post-YFV vaccination status (n = 12), or with other flaviviral (DENV = 93; WNV = 34, JEV = 25), non-flaviviral (CHIKV = 19) and *Plasmodium* spp. (PLAS: n = 69) infections. In samples from DENV-infected patients, the confirmation of DENV as the infectious agent was based on NS1 antigen detection [28]. Sera from 1,015 healthy individuals (pregnant women, blood donors and children) living in flavivirus-endemic and non-endemic areas served as negative controls. Pre-characterisation data for all control cohorts are reported in Table 2. To the best of the authors’ knowledge, none of these samples were analysed in previous studies.

**Table 2:** Characteristics of control cohorts, study evaluating a novel NS1-based ELISA, Germany 2016

Cohort	n	Origin of sample donors	Type	Diagnostic centre (provider of samples)	Sample receipt	Pre‑characterisation
**Flavivirus infection or vaccination**						
DENVa (high IgM)	47	Germany, Italy	Returning travellers from endemic areas with DENV infection (contracted e.g. in Brazil, Bali, Thailand, Laos, Philippines, India, Cambodia, Taiwan)	MVZ Diamedes GmbH Bielefeld, Germany; University of Bologna, Bologna, Italy; WHOCC, Hamburg, Germany	2011–2014	• Panbio or BIO-RAD DENV-NS1 ELISA^a,b^: 47/47 (100%) DENV-NS1 positive • DENV-RT-PCR (only 8/47 tested)^b^: n = 4 subtype DENV-1, n = 2 subtype DENV-2, n = 2 subtype DENV-3 • EUROIMMUN Anti-DENV ELISA (IgM, IgG)^c^: 40/47 (85%) anti-DENV IgM positive, 30/47 (64%) anti-DENV IgG positive, 37/47 (79%) anti-DENV IgM ratio ≥ 3.0, 10/47 (21%) anti-DENV IgM ratio < 3.0, anti-DENV IgM median ratio = 3.9
DENVb (high IgG)	46	Germany, Italy	Returning travellers from endemic areas with DENV infection (contracted e.g. in Brazil, Bali, Thailand, Laos, Philippines, India, Cambodia, Taiwan)	MVZ Diamedes GmbH Bielefeld, Germany; University of Bologna, Bologna, Italy	2011–2014	• DENV-NS1 ELISA^a,b^: 46/46 (100%) DENV-NS1 positive • DENV-RT-PCR (only 1/46 tested)^b^: n = 1 subtype DENV-4 • EUROIMMUN Anti-DENV ELISA (IgM, IgG)^c^: 35/46 (76%) anti-DENV IgM positive, 40/46 (87%) anti-DENV IgG positive, 37/46 (80%) anti-DENV IgG ratio ≥ 3.0, 9/46 (20%) anti-DENV IgG ratio < 3.0, • anti-DENV IgG median ratio = 3.9
YFV	12	France	Individuals vaccinated against YFV	Cerba Specimen Services, Saint-Ouen l'Aumône, France	2015	• YFV seroneutralisation test^d^: 12/12 (100%) anti-YFV positive • EUROIMMUN Anti-WNV ELISA (IgM, IgG)^c^: 0/12 (0%) anti-WNV IgM positive, 0/12 (0%) anti-WNV IgG positive • EUROIMMUN Anti-CHIKV ELISA (IgM, IgG)^c^: 0/12 (0%) anti-CHIKV IgM positive, 1/12 (8%) anti-CHIKV IgG positive
WNV	34	US	Patients from endemic areas with WNV infection	MAYO Clinic, Scottsdale, Arizona, US	2014	• WNV PRNT^e^: 34/34 (100%) anti-WNV positive • EUROIMMUN Anti-WNV ELISA (IgM, IgG)^c^: 23/34 (68%) anti-WNV IgM positive, 26/34 (76%) anti-WNV IgG positive
JEV	25	Vietnam	Patients from endemic areas with JEV infection	National Hospital of Tropical Disease, Hanoi, Vietnam	2016	• DRG JE IgM capture ELISA^f^: 25/25 (100%) anti-JEV IgM positive • EUROIMMUN Anti-JEV ELISA (IgM, IgG)^c^: 25/25 (100%) anti-JEV IgM positive, 19/25 (76%) anti-JEV IgG positive
**Non-flavivirus infection**						
CHIKV	19	Réunion	Patients from endemic areas with CHIKV infection	Cerba Specimen Services, Saint-Ouen l’Aumône, France	2015	• CHIKV VRP neutralisation test^g^: 19/19 (100%) anti-CHIKV positive • EUROIMMUN Anti-CHIKV ELISA (IgM, IgG)^c^: 0/19 (0%) anti-CHIKV IgM positive, 19/19 (100%) anti-CHIKV IgG positive
**Parasite infection**						
PLAS	69	France (including overseas department and region Mayotte), French Guiana, Tunisia, Madagascar, Switzerland	Blood donors living in and travellers returning from *Plasmodium*-endemic areas, acute or past *Plasmodium* infection	TheBindingSite, Schwetzingen, Germany Cerba Specimen Services, Saint-Ouen l’Aumône, France Swiss Red Cross, Bern, Switzerland	2016	• BioMérieux Plasmodium IFA (IgM, IgG)^d,h^: 1/15 (7%) anti-*Plasmodium* IgM positive, 15/15 (100%) anti-*Plasmodium* IgG positive • BIO-RAD Malaria ELISA (IgG)^i^: 54/54 (100%) anti-*Plasmodium* positive
**Healthy controls: pregnant women, blood donors and children**						
PREG	100	Germany	Pregnant women from non-flavivirus endemic areas without clinical symptoms	Laboratory Schottdorf, Augsburg, Germany	2007	• EUROIMMUN Anti-DENV ELISA (IgM, IgG)^c^: 2/100 (2%) anti-DENV IgM positive, 7/100 (7%) anti-DENV IgG positive • EUROIMMUN Anti-WNV ELISA (IgM, IgG)^c^ 3/100 (3%) anti-WNV IgM positive, 4/100 (4%) anti-WNV IgG positive • EUROIMMUN Anti-JEV ELISA (IgM, IgG)^c^: 2/100 (2%) anti-JEV IgM positive, 14/100 (14%) anti-JEV IgG positive • EUROIMMUN Anti-CHIKV ELISA (IgM, IgG)^c^: 0/100 (0%) anti-CHIKV IgM positive, 0/100 (0%) anti-CHIKV IgG positive
ZIM	128	Zimbabwe	Blood donors from flavivirus and parasite endemic areas without clinical symptoms	National Blood Transfusion Service, Zimbabwe, Africa	2003	• EUROIMMUN Anti-DENV ELISA (IgG)^c^: 4/128 (3%) anti-DENV IgG positive • EUROIMMUN Anti-CHIKV ELISA (IgG)^c^: 3/128 (2%) anti-CHIKV IgG positive • EUROIMMUN Anti-Plasmodium ELISA (IgG)^c^: 36/128 (28%) anti-*Plasmodium* IgG positive
ARG	99	Argentina	Blood donors from flavivirus endemic areas without signs of viral infection (routine samples for parasitology)	IACA Laboratory, Buenos Aires, Argentina	2014	• EUROIMMUN Anti-DENV ELISA (IgM, IgG)^c^: 2/99 (2%) anti-DENV IgM positive, 4/99 (4%) anti-DENV IgG positive • EUROIMMUN Anti-WNV ELISA (IgM, IgG)^c^: 2/99 (2%) anti-WNV IgM positive, 3/99 (3%) anti-WNV IgG positive • EUROIMMUN Anti-CHIKV ELISA (IgM, IgG)^c^: 3/99 (3%) anti-CHIKV IgM positive, 1/99 (1%) anti-CHIKV IgG positive • EUROIMMUN Anti-Trypanosoma ELISA (IgM, IgG)^c^: 2/99 (2%) anti-Trypanosoma IgM positive, 1/99 (1%) anti-Trypanosoma IgG positive
US	100	US	Blood donors without clinical symptoms (n): Hispanic (25), African American (30), Caucasian (43), Asian (1), Colombian (1)	Serologix, New Hope, Pasadena, US	2014	• EUROIMMUN Anti-DENV ELISA (IgM, IgG)^c^: 1/100 (1%) anti-DENV IgM positive, 6/100 (6%) anti-DENV IgG positive • EUROIMMUN Anti-WNV ELISA (IgM, IgG)^c^: 0/100 (0%) anti-WNV IgM positive, 4/100 (4%) anti-WNV IgG positive • EUROIMMUN Anti-CHIKV ELISA (IgM, IgG)^c^: 0/100 (0%) anti-CHIKV IgM positive, 4/100 (4%) anti-CHIKV IgG positive
GER	500	Germany	Blood donors from non-flavivirus endemic areas without clinical symptoms	University Medical Center Schleswig-Holstein, Campus Lübeck, Lübeck, Germany	2012	NA
CHIL	88	Germany	Children (≤ 10 years) form non-flavivirus endemic areas without clinical symptoms	Praxis Dr Fischer-Wassels, Dortmund, Germany	2007–2008	• EUROIMMUN Anti-DENV ELISA (IgM, IgG)^c^: 0/100 (0%) anti-DENV IgM positive, 0/100 (0%) anti-DENV IgG positive • EUROIMMUN Anti-WNV ELISA (IgM, IgG)^c^: 1/100 (1%) anti-WNV IgM positive, 0/100 (0%) anti-WNV IgG positive • EUROIMMUN Anti-JEV ELISA (IgM, IgG)^c^: 0/100 (0%) anti-JEV IgM positive, 0/100 (0%) anti-JEV IgG positive • EUROIMMUN Anti-CHIKV ELISA (IgM, IgG)^c^: 0/100 (0%) anti-CHIKV IgM positive, 0/100 (0%) anti-CHIKV IgG positive

ARG: Argentina; CHIKV: chikungunya virus; CHIL: children; DENV: dengue virus; IFA: immunofluorescence assay; GER: Germany; JEV: Japanese encephalitis virus; IIFA: indirect immunofluorescence assay; NA: not available; PLAS: *Plasmodium*; PREG: pregnant women; PRNT: plaque reduction neutralisation test; RT-PCR: reverse transcription-PCR; US: United States; WHOCC: World Health Organization Collaborating Centre (for Arbovirus and Haemorrhagic Fever Reference and Research); WNV: West Nile virus; YFV: yellow fever virus; ZIKV: Zika virus; ZIM: Zimbabwe.

^a^ Performed at MVZ Diamedis GmbH, Bielefeld, Germany.

^b^ Performed at the University of Bologna, Italy.

^c^ Performed at EUROIMMUN, Lübeck, Germany.

^d^ Performed at Cerba Specimen Services, Saint-Ouen l'Aumône, France.

^e^ Performed at the University of Leipzig, Germany.

^f^ Performed at the National Hospital of Tropical Disease, Hanoi, Vietnam.

^g^ Performed at the University of Bonn, Germany.

^h^ Performed at TheBindingSite, Schwetzingen, Germany.

^i^ Performed at the Swiss Red Cross, Bern, Switzerland.

Specimens, anamnestic/clinical information and pre-characterisation data were provided anonymised to the Institute for Experimental Immunology (affiliated to EUROIMMUN). All sera were stored at -20 °C until assayed. The study was performed according to the recommendations of the Central Ethical Committee of Germany [29].

### Enzyme-linked immunosorbent assays

Anti-Zika Virus IgM and IgG ELISA (EUROIMMUN) were used as recommended by the manufacturer. These kit assays are based on standardised reagents and microtitre plates coated with recombinant ZIKV-NS1. Briefly, sera diluted 1:101 in sample buffer were added to the wells and allowed to react for 60 min at 37 °C. Before IgM detection, sera were pre-incubated with sample buffer containing IgG/rheumatoid factor (RF) absorbent (EUROIMMUN) to remove class IgG antibodies and class IgM RF from the sample. This step prevents specific IgG from displacing IgM from the antigen (leading to false IgM-negative results) and RF-IgM from reacting with specifically bound IgG (leading to false IgM-positive results). Bound antibodies were detected by applying goat anti-human IgM peroxidase conjugate or rabbit anti-human IgG peroxidase conjugate for 30 min at room temperature, followed by staining with tetramethylbenzidine for 15 min. The enzymatic reaction was stopped by addition of one volume 0.5 mol/L sulphuric acid. A calibrator (chicken–human chimeric ZIKV antibody with a concentration adjusted to give an extinction value defining the upper limit of the reference range of non-infected persons) as well as positive and negative controls were provided with the test kit and assayed with each test run. Colour intensity of the enzymatic reactions was determined photometrically at 450 nm (reference 620 nm), resulting in extinction values. A signal-to-cut-off ratio (extinction_sample_/extinction_calibrator_) was calculated for each sample.

Receiver-operating characteristics (ROC) analysis based on the initial validation dataset of positive and negative samples was done by the manufacturer to evaluate assay performance at each possible cut-off, demonstrating optimal sensitivity and specificity at ratio values of 0.8 (IgM) and 0.6 (IgG). To ensure high specificity, the borderline range (≥ 0.8 to < 1.1) was established between the highest negative and the lowest positive validation sample, resulting in a positivity cut-off of ≥ 1.1.

Anti-dengue Virus IgM and IgG ELISA (EUROIMMUN) were used.

### Statistics

Statistical analyses were performed using GraphPad Prism 6 (GraphPad Software Inc., La Jolla, California, US) and SigmaPlot 13.0 (SSI, San Jose, California, US). Sensitivity was calculated as the proportion of ZIKV patients (referring to groups 1 to 4 as indicated) identified as positive by the assay. Specificity was calculated as the proportion of negative test results obtained among healthy controls. We calculated 95% confidence intervals (CIs) according to the modified Wald method. The study was performed in compliance with the Standards for Reporting of Diagnostic accuracy (STARD) statement [30].

## Results

### Sensitivity of the enzyme-linked immunosorbent assay

The sensitivity of the novel NS1-based anti-ZIKV ELISA was evaluated in sera from 27 patients with RT-PCR-confirmed ZIKV infection that had been sub-grouped into travellers returning from ZIKV-endemic areas and endemic-area residents. Among eight infected travellers returning from ZIKV-endemic areas (group 1), positive anti-ZIKV IgM and IgG reactivity was found in seven (87.5%) and three (37.5%) cases, respectively. Of 19 infected residents in endemic-areas (group 2), six (31.6%) were positive for anti-ZIKV IgM and 15 (79.0%) for IgG. In addition, sera from 85 patients with suspected ZIKV infection were examined. Here, of 26 infected travellers returning from ZIKV-endemic areas (group 3) 21 (80.8%) were positive for anti-ZIKV IgM and 18 (69.2%) for IgG, while among 59 infected residents in endemic-areas (group 4), six (10.2%) showed positive reactivity for anti-ZIKV IgM and 53 (89.9%) for IgG. For the total of RT-PCR-confirmed and suspected cases, the combined ELISA sensitivity (IgM and/or IgG) amounted to 23/27 (85.2%) and 78/85 (91.8%), respectively.

Confining the time point of serological evaluation to the active and late phase of ZIKV infection, i.e. ≥ 6 days after symptom onset, anti-ZIKV IgM reactivity was observed in 10/17 (58.8%) patients with positive ZIKV-RT-PCR and 3/38 (7.9%) patients with suspected ZIKV infection, while anti-ZIKV IgG was detectable in 15/17 (88.2%) and 34/38 (89.5%) cases, respectively. Thus, the combined sensitivity (IgM and/or IgG) reached 17/17 (100%) among RT-PCR-confirmed cases and 34/38 (89.5%) among suspected cases (Table 3).

**Table 3:** Anti-ZIKV reactivity in patients with RT-PCR-confirmed (n = 27) and suspected (n = 85) ZIKV infection as determined by ELISA for IgM and IgG, study evaluating a novel NS1-based ELISA, Germany 2016

Group	Characteristics		Anti-ZIKV ELISA reactivity (≥ 1 day post symptom onset)^c^				Anti-ZIKV ELISA reactivity (≥ 6 days post symptom onset)^d,e^			
			n	IgM	IgG	IgM/IgG	n	IgM	IgG	IgM/IgG
1	RT-PCR-confirmed ZIKV infection, travellers returning from ZIKV-endemic areas	Positive	8	7	3	7	5	5	3	5
		Sensitivity %^b^ (95% CI)	–	87.5 (50.8–99.9)	37.5 (13.5–69.6)	87.5 (50.8–99.9)	–	100 (51.1–100)	60.0 (22.9–88.4)	100 (51.1–100)
2	RT-PCR-confirmed ZIKV infection, residents in ZIKV-endemic areas^a^	Positive	19	6	15	16	12	5	12	12
		Sensitivity %^b^ (95% CI)	–	31.6 (15.2–54.2)	78.9 (56.1–92.1)	84.2 (61.6–95.3)	–	41.7 (19.3–68.1)	100 (71.8–100)	100 (71.8–100)
Total 1 + 2	RT-PCR-confirmed ZIKV infection	Positive	27	13	18	23	17	10	15	17
		Sensitivity %^b^ (95% CI)	–	48.1 (30.7–66.0)	66.7 (47.7–81.5)	85.2 (66.9–94.7)	–	58.8 (36.0–78.4)	88.2 (64.4–98.0)	100 (78.4–100)
3	Suspected ZIKV infection, travellers returning from ZIKV-endemic areas	Positive	26	21	18	25	NA^e^			
		Sensitivity %^b^ (95% CI)	–	80.8 (61.7–92.0)	69.2 (49.9–83.7)	96.2 (79.6–100)				
4	Suspected ZIKV infection, residents ZIKV-endemic areas	Positive	59	6	53	53	38	3	34	34
		Sensitivity %^b^ (95% CI)	–	10.2 (4.4–20.8)	89.9 (79.2–95.6)	89.9 (79.2–95.6)	–	7.9 (2.0–21.5)	89.5 (75.3–96.4)	89.5 (75.3–96.4)
Total 3 + 4	Suspected ZIKV infection	Positive	85	27	71	78	38^e^	3	34	34
		Sensitivity %^b^ (95% CI)	–	31.8 (22.8–42.3)	83.5 (74.1–90.1)	91.8 (83.7–96.2)	–	7.9 (2.0–21.5)	89.5 (75.3–96.4)	89.5 (75.3–96.4)

CI: confidence interval; NA: not available or not applicable; NS: non-structural protein; RT-PCR: reverse transcription-PCR; ZIKV: Zika virus.

^a^ This group contains 10 sera from residents of the Netherlands who were born and raised in Suriname and/or had visited their country of origin occasionally.

^b^ Referring to the total number of samples in the respective patient group during the indicated sampling period.

^c^ Referring to the whole study population of ZIKV-infected patients, i.e. samples (one per patient) taken between day 1 and day 88 post symptom onset, representing the initial (day 1–5 post symptom onset), active (day 6–20) and late phase (> 20 days) of infection.

^d^ Samples (one per patient) taken between day 6 and day 88 post symptom onset, representing the active (day 6 to 20 post symptom onset) and late phase (> 20 days post symptom onset) of infection [28].

^e^ Group 3 is not represented in the sampling period ≥ 6 days post symptom onset, because the sampling date was available for only four out of a total of 26 samples in this group.

**Figure 1:**
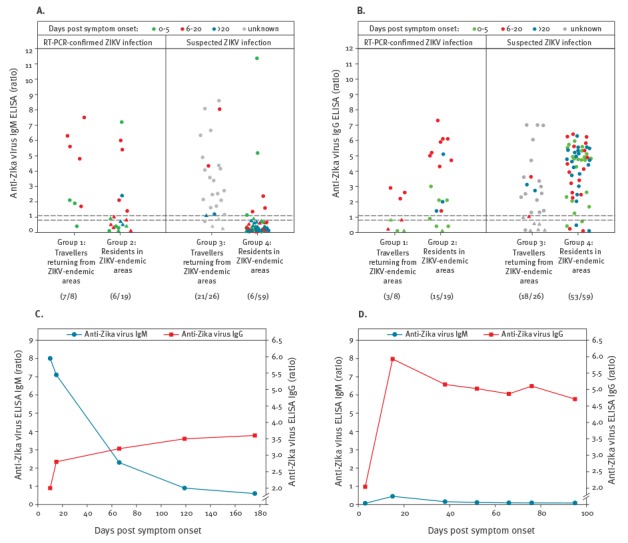
Anti-ZIKV reactivity in patients with RT-PCR-confirmed (n = 27) and suspected (n = 85) ZIKV infection as determined by ELISA for (A) IgM and (B) IgG^a^; time course analysis of anti-ZIKV antibody levels in follow-up samples from (C) a German patient returning from Colombia (probable primary ZIKV infection)^b^ and (D) a Colombian patient with RT-PCR-confirmed ZIKV infection (probable secondary flavivirus infection)^c^

RT-PCR: reverse transcription-PCR; US: United States; WHO: World Health Organization; ZIKV: Zika virus.

^a^ Per patient, one sample was examined for anti-ZIKV IgM and IgG antibodies. Plotted data points represent ratio values (extinction_sample_/extinction_calibrator_). Cut-off values for borderline results (≥ 0.8 to < 1.1) and positive results (≥ 1.1) are indicated by horizontal dashed lines. Positive and total cases are indicated in parentheses. Triangles indicate samples with a ratio for anti-ZIKV IgM or IgG below the cut-off (< 1.1), but a corresponding positive result in IgG or IgM testing, respectively.

^b^ Samples were provided by the WHO Collaborating Centre for Arbovirus and Haemorrhagic Fever Reference and Research, Hamburg, Germany. Cut-off ratio: ≥ 1.1.

^c^ Samples were provided by Biomex US LLC, Coconut Creek, Florida, US. Cut-off ratio: ≥ 1.1.

Comparing ZIKV-infected travellers returning from endemic areas (groups 1 and 3) with infected residents in these areas (groups 2 and 4), a tendency of distinct ZIKV antibody kinetics could be observed: in most returning travellers, high IgM ratio values (median 5.6; interquartile range (IQR): 4.6–6.9,) and moderate IgG ratios (median 2.2; IQR 0.9–2.8,) were detectable in the active phase of infection (cut-off ratio: 1.1). By contrast, the majority of endemic-area residents had infections with very high IgG ratios (median 4.8; IQR 3.3–5.9) during the active phase, while IgM ratios were variable, but predominantly negative or low (median 0.5; IQR 0.2–1.3) (Figure 1A and 1B).

Time course analysis of a German patient who showed clinical symptoms after returning from a stay in Colombia revealed very high anti-ZIKV IgM ratios on first testing (day 10 after symptom onset), while IgG ratios increased to moderate levels during the acute phase of infection and thereafter (Figure 1C). On the other hand, follow-up samples taken from a Colombian resident with RT-PCR-confirmed ZIKV infection indicated a significant rise in the ZIKV-specific IgG response between days 3 and 15 after symptom onset, followed by a slow decrease, while anti-ZIKV IgM was negative 3 days after symptom onset and remained below detection threshold for 14 weeks (Figure 1D).

### Cross-reactivity of the enzyme-linked immunosorbent assay

**Figure 2:**
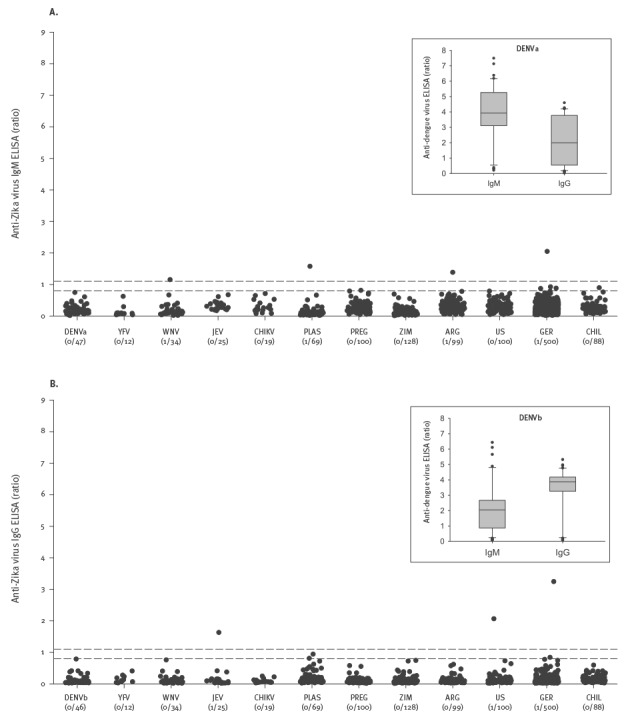
Anti-ZIKV reactivity in potentially cross-reactive samples (n = 252) and healthy controls (n = 1,015) as determined by ELISA for (A) IgM and (B) IgG^a,b^, study evaluating a novel NS1-based ELISA, Germany 2016*

ARG: Argentina; CHIKV: chikungunya virus; CHIL: children; DENV: dengue virus; GER: Germany; JEV: Japanese encephalitis virus; NS: non-structural protein; PLAS: *Plasmodium*; PREG: pregnant women; US: United States; WNV: West Nile virus; YFV: Yellow fever virus; ZIKV: Zika virus; ZIM: Zimbabwe.

^a^ Plotted data points represent ratio values (extinction_sample_/extinction_calibrator_); one data point per patient. Cut-off values for borderline results (≥ 0.8 to < 1.1) and positive results (≥ 1.1) are indicated by horizontal dashed lines. Positive and total cases are indicated in parentheses.

^b^ To provide high levels of potentially cross-reactive anti-DENV IgM and IgG antibodies, the DENV-infected patients were divided into two groups: DENVa, high median ratio (3.9) anti-DENV IgM, anti-DENV IgM ratio ≥ 3.0 in 79% of cases (inset Panel A); DENVb, high median ratio (3.9) anti-DENV IgG, anti-DENV IgG ratio ≥ 3.0 in 80% of cases (inset Panel B). Cut-off ratio (anti-DENV ELISA, EUROIMMUN): ≥ 1.1.

Cross-reactivity was analysed first in sera from 93 DENV-infected patients whose diagnosis had been secured by positive DENV-NS1 detection. This cohort was divided into one group (DENVa) with high anti-DENV IgM (median ratio 3.9) and another group (DENVb) with high anti-DENV IgG (median ratio 3.9), ensuring the presence of high levels of potentially cross-reactive antibodies. In both groups, anti-ZIKV reactivity was below the threshold, indicating absence of cross-reactivity in these specimens. Further testing, on a supplementary basis, included 159 sera from patients positive for IgM and/or IgG against YFV, WNV, JEV, CHIKV or PLAS. Anti-ZIKV IgM was positive in 1/34 (2.9%) patients infected with WNV and 1/69 (1.4%) patients infected with PLAS. Anti-ZIKV IgG was found in 1/25 (4.0%) patients infected with JEV (Figure 2). For the total of 252 potentially cross-reactive samples, the overall positivity rate amounted to 2/252 (0.8%) for IgM and 1/252 (0.4%) for IgG (Table 4).

**Table 4:** Anti-ZIKV reactivity in potentially cross-reactive specimens (n = 252) and healthy controls (n = 1,015) as determined by ELISA for IgM and IgG, study evaluating a novel NS1-based ELISA, Germany 2016

Cohort	Characteristics		Prevalence % (CI 95%)^c^		Specificity (CI 95%)^c^	
			IgM	IgG	IgM	IgG
DENVa	Dengue virus infection (high median anti-DENV IgM)^a^	47	0 (0–9.0)	0 (0–9.0)	100 (91.0–100)	100 (91.0–100)
DENVb	Dengue virus infection (high median anti-DENV IgG^b^	46	0 (0–9.2)	0 (0–9.2)	100 (90.8–100)	100 (90.8–100)
YFV	Yellow fever virus vaccination	12	0 (0–28.2)	0 (0–28.2)	100 (71.8–100)	100 (71.8–100)
WNV	West Nile virus infection	34	2.9 (0–16.2)	0 (0–12.1)	97.1 (83.8–100)	100 (87.9–100)
JEV	Japanese encephalitis virus infection	25	0 (0–15.8)	4.0 (0–21.1)	100 (84.2–100)	96.0 (78.9–100)
CHIKV	Chikungunya virus infection	19	0 (0–19.8)	0 (0–19.8)	100 (80.2–100)	100 (80.2–100)
PLAS	*Plasmodium* spp. Infection	69	1.4 (0–8.5)	0 (0–6.3)	98.6 (91.5–100)	100 (93.7–100)
Total	Potentially cross-reactive samples	252	0.8 (0–3.0)	0.4 (0–2.4)	99.2 (97.0–100)	99.6 (97.6–100)
PREG	German pregnant women	100	0 (0–4.4)	0 (0–4.4)	100 (95.6–100)	100 (95.6–100)
ZIM	Zimbabwean blood donors	128	0 (0–3.5)	0 (0–3.5)	100 (96.5–100)	100 (96.5–100)
ARG	Argentinian blood donors	99	1.0 (0–6.1)	0 (0–4.5)	99.0 (94.0–100)	100 (95.5–100)
US	US-American blood donors	100	0 (0–4.4)	1.0 (0–6.0)	100 (95.6–100)	99.0 (94.0–100)
GER	German blood donors	500	0.2 (0–1.2)	0.2 (0–1.2)	99.8 (98.8–100)	99.8 (98.8–100)
CHIL	German children	88	0 (0–5.0)	0 (0–5.0)	100 (95.0–100)	100 (95.0–100)
Total	Healthy control samples	1,015	0.2 (0–0.8)	0.2 (0–0.8)	99.8 (99.2–100)	99.8 (99.2–100)

ARG: Argentina; CHIKV: chikungunya virus; CHIL: children; DENV: dengue virus; GER: Germany; JEV: Japanese encephalitis virus; PLAS: *Plasmodium*; PREG: pregnant women; US: United States; WNV: West Nile virus; YFV: yellow fever virus; ZIKV: Zika virus; ZIM: Zimbabwe.

^a^ Median anti-DENV IgM ratio 3.9 (79% of samples with anti-DENV IgM ratio ≥ 3.0), as indicated in the inset of Figure 2A.

^b^ Median anti-DENV IgG ratio 3.9 (80% of samples with anti-DENV IgG ratio ≥ 3.0), as indicated in the inset of Figure 2B.

^c^ Referring to the total number of samples in the individual cohorts.

### Specificity of the enzyme-linked immunosorbent assay

Assay specificity was assessed by testing 1,015 sera from healthy controls. Only 1/99 (1.0%) Argentinian and 1/500 (0.2%) German blood donors were found anti-ZIKV IgM positive, while all 128 Zimbabwean and 100 US American blood donors as well as 100 German pregnant women and 88 children in Germany were negative. Anti-ZIKV IgG was present in 1/100 (1.0%) US American and 1/500 (0.2%) German blood donors, but absent in the cohorts of Zimbabwean and Argentinian blood donors, pregnant women and children. Thus, overall specificity amounted to 99.8% for either Ig class (Table 4, Figure 2).

## Discussion

The serological diagnosis of ZIKV infections has been challenging due to cross-reactions with other flaviviruses, secondary infections and previous vaccinations, which complicate interpretation, sometimes leading to unreliable or false-positive results [6,31,32]. Here, we evaluated a newly-developed ELISA with recombinant ZIKV-NS1 protein as solid-phase antigen. Huzly et al. recently provided evidence that this assay is highly specific, as demonstrated on a limited number of European patients with DENV, YFV, tick-borne encephalitis virus (TBEV) or hepatitis C virus infection [27]. In the present study, testing on specimens collected ≥ 6 days after onset of symptoms (i.e. after the viraemic phase) revealed a combined sensitivity (IgM/IgG) of 100% for RT-PCR-confirmed cases of ZIKV infection at 99.8% specificity. Among suspected ZIKV cases, the combined sensitivity amounted to 89.5%. Notably, we included only one serum sample for each of the studied patients in our analysis, except for the time course analysis. For the serological diagnosis of patients, however, the evaluation of follow-up samples is important and recommended to demonstrate seroconversion or a 4-fold increase in antibody titre [28]. In four of 27 RT-PCR-confirmed ZIKV cases, samples were negative for both IgM and IgG against ZIKV-NS1, presumably because all of them were taken only ≤ 4 days after symptom onset, i.e. when antibodies had not yet reached detectable levels. Among 85 suspected ZIKV patients, too early sampling may account for two cases with negative IgM and IgG, while the remaining five double-negative cases could be due to the absence of ZIKV infection (deficits in pre-characterisation) or to false-negative results.

Cross-reactivity with high-level DENV antibodies was not detectable and, according to preliminary analysis with a limited amount of samples, there was no indication for DENV serotype-dependent differences in cross-reactivity (data not shown). To better judge assay performance in endemic areas, samples from endemic residents who experienced multiple DENV (and other flavivirus) infections should be included in further assessments, as these samples have a potential for increased cross-reactivity. Future studies should also address a comparison of cross-reactivity with acute vs convalescent anti-DENV-positive samples, considering that the extent of cross-reactivity may be influenced by the level of circulating DENV-NS1 antigen-binding DENV-NS1 antibodies. Analysis of all potentially cross-reactive specimens resulted in positive rates of 0.8% (IgM) and 0.4% (IgG) caused by one case each with WNV and PLAS with low-level anti-ZIKV IgM and one JEV case with low-level anti-ZIKV IgG. In these cases, however, double infections cannot be excluded, so it remains unclear if ELISA positivity resulted from the presence of ZIKV antibodies due to co-infection with ZIKV (true-positive) or from cross-reactivity (false-positive). In case of PLAS infection, PLAS-induced polyclonal B-cell activation may cause the production of potentially cross-reactive antibodies [33]. Among patients with current PLAS infection, up to 30% false-positive or borderline reactions were reported using the presented NS1-based ELISA [34], which is in contrast to only 1.4% in the present study and probably explained by the fact that our cohort was comprised mainly of individuals with past PLAS infection status. Possible interferences should thus be considered when applying the assay.

In sera from travellers returning from ZIKV-endemic areas, we observed a tendency of ZIKV-specific IgM to appear at high ratios during the active phase of infection, paralleled by a moderate rise in IgG. In contrast, most residents in endemic areas had high anti-ZIKV IgG and low/negative IgM ratio values, irrespective of whether their samples were taken during the initial, active or late phase of infection. IgM responses in travellers returning from ZIKV-endemic areas tended to be higher compared with residents in such areas, whereas the IgG-positivity rate was higher in the latter subgroup. Such differences in ZIKV antibody kinetics were also illustrated by time course analysis of antibody levels in two representative patients, possibly reflecting that travellers returning from ZIKV-endemic countries predominantly had a primary flavivirus/ZIKV infection, while most residents probably contracted ZIKV as a secondary flavivirus infection. Similar kinetics have been described for primary and secondary infections in the Micronesian ZIKV epidemic [6] and for DENV-infected patients [35,36], suggesting that the detection of both specific IgM and IgG is diagnostically important and relevant for differentiating primary from secondary infections. Regarding our comparison of patients residing in endemic countries vs travellers, however, systematic differences in the background of these populations (e.g. genetic, ethnic) cannot be excluded.

Another limitation of our study is that it does not comprise side-by-side testing with additional assays, such as the Zika MAC-ELISA (Centers for Disease Control and Prevention (CDC), Atlanta, Georgia, US) or PRNT, to provide comparative data on these current tests. In addition, the non-deliberate absence of a uniform serological reference standard for the pre-characterisation of all ZIKV samples resulted in a high number of suspected cases of ZIKV infection.

Although ZIKV usually causes rather mild infections, there has been convincing evidence of a causal link to neuronal impairment, such as newborn microcephaly and GBS [37]. Furthermore, there have been studies showing that DENV NS1 antibodies have the potential of inducing autoantibodies in secondary infections, probably mediated by cross-reactive binding of antigens on platelets and endothelial cells, followed by cellular damage and inflammatory activation [17]. Basic research is needed to fully elucidate the causal relations between neuronal disorders and ZIKV infection. Epidemiologic assessment of pregnant women and their babies, and of travellers returning from endemic areas, the surveillance of donated blood and the investigation of ZIKV prevalence in endemic and non-endemic areas may provide crucial information. These studies need reliable, fast, and easy-to-handle diagnostic tests that have low cross-reactivity and allow a definite diagnosis.

In conclusion, our study revealed that the NS1-based anti-ZIKV ELISA is a sensitive and highly specific tool for the serodiagnosis of ZIKV infections, eliminating cross-reactions with antibodies to DENV and other flaviviurses. The assay format is suitable for use in routine laboratories worldwide enabling high-throughput testing in epidemic settings. Serological identification of ZIKV infections is maximised by parallel testing for IgM and IgG. Further studies will be necessary to determine the accuracy of this and other current assays in a larger set of well-defined samples, and to clarify how ZIKV infection triggers GBS, newborn microcephaly and other neurological manifestations.

## *Erratum

The title of the y-axis in Figure 2B was corrected to read ‘Anti-Zika virus IgG ELISA (ratio)’ on 22 December 2016.
