# Erratum for Euro Surveill. 2016;21(50)

**DOI:** 10.2807/1560-7917.ES.2017.22.1.30430

**Published:** 2017-01-05

**Authors:** Eurosurveillance editorial team

**Affiliations:** 1 European Centre for Disease Prevention and Control (ECDC), Stockholm, Sweden

In the article ‘Serodiagnosis of Zika virus (ZIKV) infections by a novel NS1-based ELISA devoid of cross-reactivity with dengue virus antibodies: a multicohort study of assay performance, 2015 to 2016’ by K Steinhagen et al., the title of the y-axis in Figure 2B was corrected to read ‘Anti-Zika virus IgG ELISA (ratio)’ on 22 December 2016.

